# Electronic Cigarette Use and Myocardial Infarction

**DOI:** 10.7759/cureus.48402

**Published:** 2023-11-06

**Authors:** Talal Alzahrani

**Affiliations:** 1 Internal Medicine Department, College of Medicine, Taibah University, Madinah, SAU

**Keywords:** health public, nhis, epidemiology, myocardial infarction, electronic cigarettes

## Abstract

Background: Current electronic cigarettes (e-cigarettes) is associated with myocardial infarction, controlling whether the subjects smoke cigarettes. However, no studies have been conducted on subjects who never smoked cigarettes. This study aimed to determine the association between e-cigarette use and myocardial infarction among subjects who have never smoked cigarettes.

Methods: The National Health Interview Survey (NHIS) data from 2014 to 2021 was used to evaluate the relationship between e-cigarette use and myocardial infarction in subjects who have never smoked cigarettes after adjusting for risk factors, including age, sex, diabetes, hypertension, hypercholesterolemia, and obesity/overweight, using logistic regression.

Results: A total of 139,697 subjects were never users, and 1,237 subjects were current e-cigarette users. E-cigarette users were significantly younger than never users. E-cigarette users were less likely to be female (40% vs. 60%, p <0.01), or have diabetes (3% vs. 10%, p <0.01), have hypertension (11% vs. 32%, p <0.01), have hypercholesterolemia (8% vs. 27%, p <0.01), or be overweight or obese (56% vs. 65%, p <0.01) compared to never users. The current e-cigarette users had a 2.6-fold increase in the odds of having a myocardial infarction (OR 2.62, 95% CI 1.44-4.77; p <0.01) after adjusting for sex, age, hypertension, diabetes, hypercholesterolemia, and obesity/overweight.

Conclusions: This study suggests that current e-cigarette use may increase the risks of myocardial infarction in subjects who never smoked cigarettes. Further longitudinal studies are needed to confirm the results of this study.

## Introduction

Electronic cigarettes (e-cigarettes) are devices that vaporize liquid nicotine, which the user then inhales. E-cigarettes are often promoted as a safe alternative to traditional cigarettes, but there is growing evidence that they may pose serious health risks [1,2]. E-cigarette use has increased dramatically in the last decade and has become an epidemic risk behavior, especially among teenagers and young adults [3-5]. In the United States, the prevalence of e-cigarette use among high school students increased from 1.5% in 2011 to 27.5% in 2019 [6]. E-cigarette use leads to several biological changes associated with an increased risk of cardiovascular disease. E-cigarette use inhibits endothelial function by reducing nitric oxide production from endothelial cells and increasing reactive oxygen species production, which leads to endothelial cell death [7]. Consistent with these observations, e-cigarette exposure inhibits arterial flow-mediated dilation to the same extent as exposure to conventional cigarette smoke [8]. E-cigarette use has pro-inflammatory effects by increasing oxidative stress and inflammatory markers such as tumor necrosis factor-α, interleukin-8, interleukin-6, and interleukin 1β [9-12]. Lee et al. found that acute exposure to flavored e-cigarette liquid leads to endothelial dysfunction by decreasing cell viability and increasing the level of reactive oxygen species [12]. This biological study also showed that exposure to e-cigarette liquids activates macrophage cells and increases cytokine production, such as interleukin-1β and -6, which are known to be associated with atherosclerotic coronary artery disease [13]. E-cigarette use has been associated with increased platelet activation and aggregation in both conventional and e-cigarette smokers who have never smoked cigarettes [14-16]. Several recent studies have shown a link between e-cigarette use and cardiovascular disease, independent of the effects of former or current cigarette smoking [17-20]. While these studies controlled for cigarette smoking (which was shown to be a statistically independent risk factor from smoking cigarettes) and included subjects who only used e-cigarettes, concerns have been expressed that the observed associations between e-cigarette use and cardiovascular disease could be a residual effect of former smoking or due to smokers switching to e-cigarettes following having a myocardial infarction [18,21,22]. This study avoids those concerns by examining the association between e-cigarette use and cardiovascular disease in subjects who never smoked conventional cigarettes.

## Materials and methods

Study population

The National Health Interview Survey (NHIS) is a large-scale survey that collects health data among adults in the United States. The NHIS provides valuable information that can be used to improve public health [23]. Data from 2014 to 2021 was used in this study to evaluate the association between myocardial infarction and e-cigarette use in subjects who never smoked conventional cigarettes. Specifically, any respondent who responded “no” to " have you smoked at least 100 cigarettes in your entire life?” was included in this study, n=148,837 subjects. 

Dependent variable (outcome) 

Myocardial Infarction (MI)

Subjects who responded “yes” to “(Ever told) you had a heart attack, also called a myocardial infarction?” were considered to have a myocardial infarction. 

Exposure of interest

E-Cigarette Use

Subjects who responded, “Some days” or “Every day” to “Do you now use e-cigarettes or other electronic vaping products every day, some days, or not at all?” were classified as “current e-cigarette users.” 

Demographic Covariates

Sex and age were collected during the survey. These data were collected by asking the subjects, “What was your sex at birth? Was it male or female?” and Age as a continuous variable. 

Clinical Covariates

We considered subjects having the risk factors of cardiovascular disease such as diabetes, hypercholesterolemia, and hypertension based on the answers to the questions “(Ever told) (you had) diabetes?” and “Have you ever been told by a doctor, nurse, or other health professional that...” for “high blood pressure” and “high cholesterol.” Subjects who responded “no” were coded as “no,” subjects who answered “yes” were coded as “yes,” and other subjects were labeled as missing. Adults with a body mass index ≥25 were labeled obese/overweight. 

Statistical analysis

The baseline demographic and clinical characteristics were reported as a percentage for categorical variables and a median for continuous variables. A chi-square test was conducted for categorical variables to compare the two groups (current e-cigarette users vs. never users). The Wilcoxon Rank Sum Test was used to compare the differences in age between the two groups because age was not normally distributed based on the Shapiro-Wilk test (p <0.01). 

We used logistic regression models to calculate the odds of myocardial infarction as a function of e-cigarette use to calculate the odds ratio of having these diseases after adjusting for cardiovascular risk factors, including age, sex, diabetes, hypertension, hypercholesterolemia, and overweight/obesity. Analyses were performed using STATA version 16. All tests used α=0.05 as the probability for a Type I error.

## Results

The demographic and health characteristics of the subjects are shown in Table 1. A total of 139,697 subjects were never users, and 1,237 subjects were current e-cigarette users. The data analysis showed that current e-cigarette users were significantly younger than never users. E-cigarette users were less likely to be female (40% vs. 60%, p <0.01), or have diabetes (3% vs. 10%, p <0.01), have hypertension (11% vs. 32%, p <0.01), hypercholesterolemia (8% vs. 27%, p <0.01), have or be overweight or obese (56% vs. 65%, p <0.01) compared to never users.

**Table 1:** The differences in demographic and health characteristics between current e-cigarette users and never users

Variable	E-cigarette use		P-value
	Never users % (n)	Current users % (n)	
Sample size	99% (139,697)	1% (1,237)	
Demographics			
Women	60% (83,882)	40 % (500)	<0.01
Age, year (median)	50	28	<0.01
Health Status			
Hypertension	32% (44,432)	11% (140)	<0.01
Diabetes Mellitus	10% (13,430)	3% (37)	<0.01
High Cholesterol	27% (38,228)	8% (93)	<0.01
Obesity/Overweight	65% (90,164)	56% (688)	<0.01

Cardiovascular disease outcomes

The odds ratio for myocardial infarction is shown in Table 2. The current e-cigarette users had a high odds ratio of myocardial infarction. The current e-cigarette users had a 2.6-fold increase in the odds of having a myocardial infarction (OR 2.62, 95% CI 1.44-4.77; p <0.01) after adjusting for sex, age, hypertension, diabetes, hypercholesterolemia, and obesity/overweight. 

**Table 2:** Multivariable associations between e-cigarette use and myocardial infarction of NHIS 2014-2021 combined

Model	Model 1*		Model 2†	
Characteristics	OR (95% CI)	P-value	OR (95% CI)	P-value
E-cigarette use	2.30 (1.28-4.13)	<0.01	2.62 (1.44-4.77)	<0.001
Women	0.44 (0.41-0.47)	<0.01	0.44 (0.41-0.48)	<0.01
Age	1.07 (1.07-1.08)	<0.01	1.06 (1.06-1.06)	<0.01
Hypertension			2.20 (2.01-2.41)	<0.01
Diabetes Mellitus			1.88 (1.73-2.04)	<0.01
High Cholesterol			2.11 (1.95-2.29)	<0.01
Obesity/Overweight			1.16 (1.07-1.27)	<0.01
*Model 1 adjusted for age and sex. †Model 2 adjusted for all demographic and health characteristics.				

Age (OR 1.07, 95% CI 1.07-1.08; p <0.01), hypertension (OR 2.20, 95% CI 2.01-2.41; p <0.01), diabetes (OR 1.88, 95% CI 1.73-2.04; p <0.01), hypercholesterolemia (OR 2.11, 95% CI 1.95-2.29; p <0.01), and obesity/overweight (OR 1.16, 95% CI 1.07-1.27; p <0.01) were associated significantly with myocardial infarction.

## Discussion

This study detected a significant relationship between e-cigarette use and myocardial infarction among subjects who never smoked conventional cigarettes. The results of this study suggest that current e-cigarette use may be associated with myocardial infarction. This finding is consistent with previous studies showing a link between e-cigarette use and cardiovascular risk factors, such as inflammation and oxidative stress [9-12]. This study also suggests that the increased odds of myocardial infarction associated with e-cigarette use are not simply due to demographic or health characteristics differences between current e-cigarette users and never users.

Osei et al. (19) used the 2016 and 2017 Behavioral Risk Factor Surveillance System data. They found no significant association between current e-cigarette use and cardiovascular disease (OR 1.04, 95% CI 0.63-1.72) in never-smokers. However, they did find a significant increase in risk associated with e-cigarette use among smokers compared to those who smoke conventional cigarettes, which suggests that e-cigarette use is associated with cardiovascular disease. Compared to the study of Osei et al., our analysis detected an association between e-cigarette use and myocardial infarction among subjects who never smoked conventional cigarettes. Our study was able to detect this association for several reasons. First, it used a large dataset of eight years (2014-2021). Second, the study examined the association between e-cigarette use and myocardial infarction in subjects who never smoked conventional cigarettes, avoiding any possible bias associated with tobacco smoking. Third, the study used a rigorous analysis to control for confounding factors. Our study showed that current e-cigarette users were very young, which made them less likely to have cardiovascular risk factors and myocardial infarction. Therefore, it is difficult to establish the association between e-cigarette use and myocardial infarction among this group without having a large study with reasonable control for cardiovascular risk factors. 

Since we published the first study, which showed daily e-cigarette use is associated with myocardial infarction independent of the effects of tobacco smoking [17], several researchers have argued that the association between e-cigarette use and myocardial infarction could be biased by smokers who switched to e-cigarettes following having a myocardial infarction [21,22], even though smokers who developed myocardial infarction are less likely to use e-cigarettes following their myocardial infarction than subjects who did not have a myocardial infarction [18,24,25]. The present study avoids any possible bias associated with tobacco smoking by examining the association between e-cigarette use and myocardial infarction in subjects who never smoked conventional cigarettes. The results of this study are consistent with biological studies, which showed that e-cigarette use is associated with endothelial dysfunction, oxidative stress, inflammation, and platelet activation [7, 9-12,14-16]. 

Our study showed that most current e-cigarette users were between 18 and 24. These findings raise another concern regarding the future trend in the incidence of cardiovascular disease in young individuals who use e-cigarettes. E-cigarettes should not be recommended, and current users of e-cigarettes should be urged to stop using all tobacco products, including e-cigarettes.

Limitations

This study has several limitations. The cross-sectional design of this study means that it can only identify associations, not causal relationships. Propensity score analysis was used in this study to reduce confounding variables but cannot be used to establish causal inference. To establish causal inference, it is necessary to have a longitudinal study design. Additionally, the study relies on self-reported data, subject to recall and misclassification biases. However, the BRFSS survey asks respondents if they have been told by a doctor, nurse, or other health professional that they have a heart attack, coronary heart disease, or stroke. This question is designed to help reduce recall bias by asking respondents to report on a medical diagnosis made by a healthcare professional. In addition, studies in Finland and Minnesota found a high degree of agreement between self-reported myocardial infarctions and medical records. This suggests that the NHIS survey is a reliable data source on cardiovascular disease [26,27].

We acknowledge that it is not known when cardiovascular disease started relative to e-cigarette use in the study subjects. It is possible that some of the subjects with cardiovascular disease reported that they had the illness before e-cigarettes became available in the US in 2007. We tried to exclude those individuals from their analysis, but there was not enough data on when the subjects first developed heart problems, so this was impossible. This situation means the study's odds ratio (OR) estimates are biased towards the null. This means that the study's results likely underestimate the risks associated with e-cigarette use [14].

The results may be confounded by factors not included in the analysis. For example, the study did not collect data on the subjects' family history of cardiovascular disease, medication use, exercise, or obstructive sleep apnea. This study did not consider the potential variation in e-cigarette brands. E-cigarette brands can contain different ingredients, and these ingredients can have varying health effects. These factors could influence the relationship between e-cigarette use and cardiovascular disease. In addition, the study did not assess the dose-response relationship between e-cigarette use and myocardial infarction. This means the study did not examine how the risk of myocardial infarction changes as e-cigarette use increases. More research is needed to confirm the study's findings and assess the dose-response relationship between e-cigarette use and myocardial infarction.

## Conclusions

E-cigarettes may be associated with myocardial infarction in subjects who have never smoked cigarettes. The findings of this study suggest that it is essential to raise awareness of the potential risks of e-cigarettes, especially among youth and young adults. Longitudinal research is needed to confirm the results of our study. 
