# Correction: Electronic Cigarette Use and Myocardial Infarction

**DOI:** 10.7759/cureus.c164

**Published:** 2024-03-07

**Authors:** Talal Alzahrani

**Affiliations:** 1 Internal Medicine Department, College of Medicine, Taibah University, Madinah, SAU

This article has been corrected by the Editor-in-Chief. The online and PDF versions have been updated accordingly. The corrections are as follows:

1. The following sentence in the Abstract has been corrected as follows: "This study suggests that current e-cigarette use increases the risks of cardiovascular disease, including myocardial infarction and stroke, in subjects who never smoked cigarettes." corrected to "This study suggests that current e-cigarette use may increase the risks of myocardial infarction in subjects who never smoked cigarettes."
2. The following sentence in the Discussion section has been corrected as follows: "The results of this study suggest that current e-cigarette use is associated with myocardial infarction." corrected to "The results of this study suggest that current e-cigarette use may be associated with myocardial infarction."
3. The following sentence in the Conclusions section has been corrected as follows: "E-cigarettes are associated with myocardial infarction in subjects who have never smoked cigarettes." corrected to "E-cigarettes may be associated with myocardial infarction in subjects who have never smoked cigarettes."
4. Reference 18 has been updated to note that the referenced article has been retracted: Bhatta DN, Glantz SA: Electronic cigarette use and myocardial infarction among adults in the US population assessment of tobacco and health. J Am Heart Assoc. 2019, 8:e012317. 10.1161/JAHA.119.012317
5. The paragraph following Table 2 has been corrected so that the odds ratios match those listed in Table 2.

The author agrees with this correction.

